# Retzius-Sparing Robot-Assisted Radical Prostatectomy Using the Hinotori Surgical Robot System Platform: Report of the First Series of Experiences

**DOI:** 10.3390/curroncol31090410

**Published:** 2024-09-17

**Authors:** Yuta Yamada, Shigenori Kakutani, Yoichi Fujii, Naoki Kimura, Yuji Hakozaki, Jun Kamei, Satoru Taguchi, Aya Niimi, Daisuke Yamada, Haruki Kume

**Affiliations:** Department of Urology, Graduate School of Medicine, The University of Tokyo, Tokyo 113-8655, Japan; kakutanis-uro@h.u-tokyo.ac.jp (S.K.); fujiiy-uro@h.u-tokyo.ac.jp (Y.F.); naoki.kimura331@gmail.com (N.K.); hakozakiy-tra@h.u-tokyo.ac.jp (Y.H.); kameij-uro@h.u-tokyo.ac.jp (J.K.); taguchis-uro@h.u-tokyo.ac.jp (S.T.); niimia-uro@h.u-tokyo.ac.jp (A.N.); yamadai77777@gmail.com (D.Y.); kumeh-uro@h.u-tokyo.ac.jp (H.K.)

**Keywords:** hinotori, Retzius-sparing robot-assisted radical prostatectomy, rs-RARP, radical prostatectomy

## Abstract

Background: The aim of this study is to describe the first series of six patients undergoing Retzius-sparing robot-assisted radical prostatectomy (rs-RARP) using the hinotori surgical robot system (hinotori SRS) and to compare the treatment outcomes with those achieved with the da Vinci surgical platform. Methods: This study included 20 cases involving the rs-RARP procedure (hinotori: N = 6; da Vinci: N = 14) that were performed between May 2021 and April 2024 in a single institution. Results: No significant differences were observed between the hinotori and da Vinci groups regarding the preoperative findings. In the hinotori group, there were four cases of pT2 that showed negative surgical margins in all the cases. However, positive surgical margins were observed in two of the cases with pT3. The surgical outcomes were also similar between the two groups except for console time, which tended to be shorter in the da Vinci group (*p* = 0.058). There were no major complications in the initial six cases with the hinotori SRS. Immediate urinary continence was observed in 50% of the cases with the hinotori group compared with 64% for the da Vinci group. Conclusion: This is the first study to report cases of rs-RARP performed on a hinotori SRS. It seems that the hinotori SRS shows similar treatment outcomes compared with the cases treated via the da Vinci platform.

## 1. Introduction

Retzius-sparing robot-assisted radical prostatectomy (rs-RARP) was first introduced by Galfano A. in 2010 [1]. This novel approach is unique since it is not associated with the dissection of the Retzius space, providing excellent early recovery of urinary continence [2]. However, this technique is associated with a small working space, which makes this procedure very difficult to perform.

The da Vinci surgical system (Intuitive Surgical, Sunnyvale, CA, USA) has been the central player in robot-assisted surgeries for over two decades. However, new types of robotic platforms have been introduced in the market, one of which is the hinotori SRS that was developed by Medicaroid Corporation (Kobe, Japan) in 2019. Other surgical robotic systems include Saroa (Riverfiled Riverfield Inc., Tokyo, Japan) and the Hugo RAS System (Medtronic, Minneapolis, USA). Saroa is unique since it provides a sense of touch to surgeons by using the air pressure of the pneumatic control system. The Hugo RAS System is composed of individual arm configurations that enable flexibility in the range of motion. The hinotori SRS was designed very similarly to the da Vinci surgical system, which has four robotic arms, no sense of touch, and a similar type of forceps. This surgical platform has arms possessing eight axes that contribute to more flexible movement and prevent interference between arms [3]. It is also implemented without trocar docking, unlike the docking system of the da Vinci platform. This provides a clear-out space around the trocar, which enables the assistant surgeon to see the patient’s abdominal surface.

This study reports the first experience of rs-RARP using the hinotori SRS and its oncologic and functional outcomes compared to those with the da Vinci platform. It is also the first report to show the actual performance of this platform by video clips.

## 2. Materials and Methods

### 2.1. Patients and Study Design

A total of 6 and 14 patients underwent rs-RARP using the hinotori SRS and da Vinci surgical system between May 2021 and April 2024 in a single institution. The two groups were compared in terms of perioperative clinical and pathological parameters. Age, prostate-specific antigen, body mass index, prostate volume, and clinical T stage were investigated for preoperative parameters. Regarding perioperative parameters, console time, estimated blood loss, nerve sparing status, prostate weight, surgical margin status, pathological T status, complications, and functional outcomes (immediate urinary continence and sexual function) were investigated. Immediate urinary continence was defined as “using no pads within 24 h after surgery”, and immediate erectile functional recovery was defined as “patient claim of erection or score of more than 0 on Erectile Hardness Score within 24 h after surgery”. This study was a retrospective study.

Written informed consent was obtained from all study participants, and this study was approved by the ethics committee “Research Ethics Committee of the Faculty of Medicine of the University of Tokyo” (ID: #3124) of the University of Tokyo. This study was conducted under the Helsinki Declaration.

### 2.2. Surgical Method

**Figure 1:**
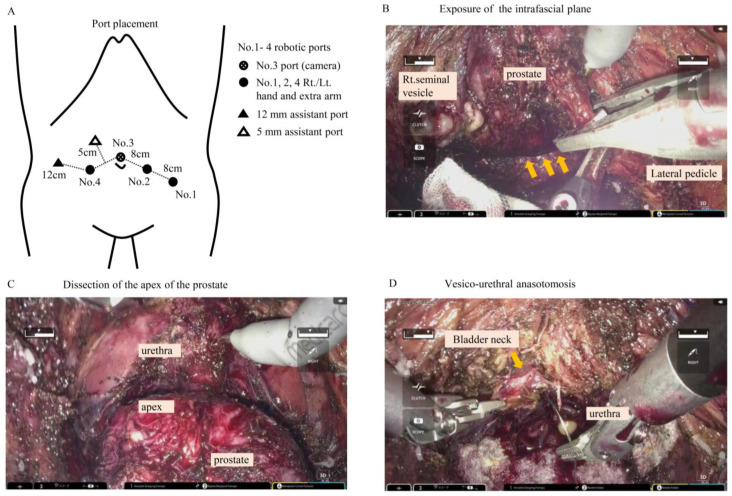
(**A**) Port placement in rs-RARP procedure. RARP: robot-assisted radical prostatectomy. (**B**) Exposure of intrafascial plane. The orange arrow shows the intrafascial plane. The assistant is using a clip to dissect the lateral pedicle. (**C**) Dissection of the apex of the prostate. (**D**) Vesico-urethral anastomosis. The orange arrow shows the bladder neck. Note that the bladder neck reconstruction was performed using 4-0 poly-absorbable filament (everting technique).

RARP was performed by either using the hinotori Surgical System ™ (Medicaroid, Kobe, Japan) or the da Vinci surgical robot system (da Vinci-S, Si, or Xi ^®^: Intuitive Surgical Incorporation, Sunnyvale, CA, USA). RARP was performed in a lithotomy-Trendelenberg position (25 degrees). The RARP procedure was carried out by a transperitoneal approach using 6 ports, 4 of which were for robotic arms (Figure 1A).

Briefly, the steps of the procedure were as follows:

1. Dissection of the peritoneum above the Douglas pouch (Video S1). The peritoneum was cut widely across the pelvis.
2. Identifying and dissecting vas deferens and seminal vesicles (Video S2).
3. Cutting the Denonvillier’s fascia and dissecting the intrafascial plane (Figure 1B).
4. Spreading the surface of the intrafascial or interfascial plane around the prostate (Video S3).
5. Dissection and isolation of the bladder neck: Bladder neck preservation was conducted in all cases (Video Clip S3).
6. Bladder neck reconstruction: Performing a plastic procedure on the bladder neck using the everting technique (Video Clip S4). Suture with 4-0 poly-absorbable filament is placed in 3–4 directions, usually at 0, 3, 9, and 6 o’clock.
7. Dissection of the apex (Figure 1C).
8. Cutting the urethra. The urethra was cut adjacent to the distal edge of the prostatic apex to preserve the urethra as much as possible.
9. Resection of the entire prostate.
10. Hemostasis.
11. Transection of the urethra: The urethra was cut adjacent to the distal edge of the prostatic apex.
12. Urethro-vesico anastomosis using the Van Velthoven technique [4]. This procedure was carried out by a single-knot running suture of 3-0 absorbable monofilament (Figure 1D).
13. Leak check.

All 20 surgeries were performed by a single surgeon (Y.Y.). The surgical procedure is based on the report by Galfano et al. [1,2], except that the patients were placed in a 25-degree Trendelenburg position and the non-barbed type of suture was used in urethro-vesico anastomosis. This procedure was completely the same when using hinotori SRS or the da Vinci. A 30-degree lens was used throughout the surgery. Trocars were placed in a semi-lunar arrangement as shown in Figure 1A. The surgery started off by the dissection of the peritoneum above the Douglas pouch. The dissection line was extended along the vas deferens. Careful dissection is required so as not to injure the ureter. After vas deferens and the seminal vesicle were identified, dissection of the posterior part of prostate was performed. Intrafascial plane was exposed after the cold cut resection of the Denonvillier’s fascia. By extending this plane towards the corner of the postero-lateral part of the prostate, the dissection moves anterior to the prostate. Bladder neck preservation technique was used in all the cases. Everting suture using the 4-0 poly-absorbable filament was performed following the bladder neck transection. The anterior part and the apex of the prostate were dissected. Urethra was cut so as to preserve urethral length as much as possible. Dissection was performed around the prostate on all sides to resect the entire prostate. Urethro-vesico anastomosis was performed using a single-knot (2 needle threads tied at the center to make a single knot) 3-0 absorbable monofilament. Saline (100 mL) was infused into the bladder via urethral catheter to check watertight anastomosis.

### 2.3. Statistical Analyses

Statistical analysis was carried out using the statistical software JMP^®^ PRO17.0.0. Wilcoxon rank-sum test was used to compare differences in continuous values between the 2 groups. The chi-squared test and Fisher test were used for the analysis of categorical values. A *p*-value of <0.05 was considered statistically significant.

## 3. Results

Table 1 shows the clinical and surgical outcomes of the patients undergoing rs-RARP. The median values (hinotori vs. da Vinci) of the preoperative clinical parameters were not statistically significant, including age, prostate-specific antigen (PSA), body mass index (BMI), and cT stage. 

The postoperative parameters are also shown in Table 1. There was a tendency for shorter console time in the da Vinci group (*p* = 0.058). There were no other significant differences between the two groups. Notably, the positive surgical margin rates were 0% and 16.7% in the hinotori and da Vinci groups, respectively. A Clavien–Dindo grade 3 complication was observed in one patient in the da Vinci group who had an impacted urethral stone postoperatively. Regarding urinary continence, 50% and 64.3% of the patients achieved immediate pad-free status. All the patients in the hinotori SRS achieved 1 pad/day within 3 months as compared with 78.6% in the da Vinci group (Table 1). Immediate erectile function was not observed in any of the patients either in the hinotori SRS or in the da Vinci group.

A detailed description of the six cases performed with the hinotori SRS is shown in Table 2. Three cases were not followed sufficiently to observe the pad-free status of the 3-month postoperative period. In the hinotori group, one patient experienced neuropathy of the upper extremity postoperatively, but it resolved within 3 days of observation. Two patients recovered their erectile function within 3 months.

## 4. Discussion

In the present study, we described the initial series of six cases of rs-RARP performed with the hinotori SRS. Initially, the authors assumed that the “floating-like sensation” of the arms may influence the surgical and functional outcomes. Fortunately, the hinotori SRS showed an excellent resection margin rate for the pT2 cases and also showed similar results regarding recovery of urinary continence. Additionally, we had no unrecoverable malfunctions resulting in conversion to open or laparoscopic surgery. Although the console time was approximately 40 min longer with the hinotori SRS, this may be due to the experience of the surgeon and the assistant. Moreover, the “floating-like sensation” may have influenced the slower movement of the arms of the robotic system when compared with da Vinci.

The novel surgical platform, the hinotori SRS, was first approved for sale in Japan in 2020. Since then, the surgical outcomes of the initial series of RARP approaches, robot-assisted partial nephrectomy, robot-assisted adrenalectomy, robot-assisted radical nephrectomy, and robot-assisted nephroureterectomy, were reported and over 50 hinotori SRSs have been in use in Japan [1,5,6,7,8,9,10]. Hinata N. et al. reported the first 30 cases of RARP without having conversion to open or laparoscopic surgery [1]. Device errors occurred in four out of the thirty cases but recovered within approximately 30 min (range: 8–31 min) [1]. In our series, we had no device errors or malfunctions. Conversion to open or laparoscopic surgery was also not observed.

The merits of using the hinotori SRS are the low cost, 3D full high vision (1920 × 1080 pixels), and cleared-out space around the trocars. The hinotori platform provides smooth manipulation of the arms during the suturing procedure, as shown in our video clips. However, there are still some aspects to be improved. The “floating-like sensation” of the arms may be associated with the direct insertion of the forceps. This phenomenon is considered to be caused by the change in volume regarding the pneumoperitoneum. Since the forceps are inserted directly, there is instability when the pneumoperitoneum volume changes, leading to an abnormal sensation for the surgeon. Fortunately, this “floating-like sensation” has already been incredibly improved as of the latest update in autumn of 2023. Notably, the loosening of the cables providing the Degree of Freedom (DOF) has not been reported nor considered as the cause of this phenomenon.

Original sealing devices, staplers, and dual consoles have not yet been developed for the hinotori SRS, and this is a great disadvantage in robot-assisted radical cystectomy. However, in the rs-RARP procedure, the robotic sealing device may be altered by the assistant using the laparoscopic sealing device, and staplers are not used. Since dual consoles have also not been developed yet, the da Vinci surgical system may provide better education for second-generation surgeons. Additionally, the flipping of the 30-degree-lens camera from “downward” to “upward” or vice versa requires several steps (10–15 s), which makes it time-consuming when compared with da Vinci.

The current list price of da Vinci (Xi, single console) is JPY 276,000,000, and the double console costs JPY 334,000,000 in Japan. The maintenance fees for da Vinci are JPY 14,000,000 and JPY 17,500,00 for the single and double consoles, respectively. On the contrary, the price of the hinotori SRS is JPY 235,000,000. Fortunately, the retail price of the hinotori SRS is often much lower, which makes it attractive to potential users. The maintenance fee of the hinotori SRS is JPY 13,000,000, which is similar to the da Vinci single console. The low cost of the hinotori SRS may be attractive to relatively smaller hospitals and hospitals that already have multiple surgical robots. 

In a propensity-score-matched analysis, the hinotori SRS and da Vinci showed similar surgical outcomes regarding the conventional RARP [11]. On the other hand, the rs-RARP procedure is almost completely different from the conventional RARP procedure, and it is associated with a very small space, and, often, it is difficult for the surgeon to perform dissection around the prostate. Nevertheless, our present study indicated that the hinotori platform showed acceptable performance and similar surgical outcomes compared with the da Vinci platform. 

This study has some limitations. Given the small number of recorded cases, it may be premature to draw definitive clinical conclusions. However, in the initial six cases, the hinotori SRS demonstrated performance comparable to that of the da Vinci system. Moreover, the retrospective nature of the study may further limit drawing conclusions.

## 5. Conclusions

We described the first series of rs-RARP cases using the hintori SRS. It seems that this surgical platform is feasible to be implemented in the rs-RARP procedure. More extensive research is required to further assess the performance of this newly developed surgical system.

## Figures and Tables

**Table 1:** Clinical and surgical outcomes comparing the 2 robotic platforms.

		Hinotori	Da Vinci	*p* Value
Preoperative Parameters		Median Value or Cases		
Age		69	63	0.231
PSA (ng/mL)		6	6.7	0.433
BMI (kg/m^2^)		21.7	23.2	0.149
Prostate volume (cm^3^)		35.3	31.9	0.43
cT stage	cT2	6	14	1.000
	cT3	0	0	
**Postoperative parameters**				
Console time (min.)		287	242	0.058
EBL (mL)		225	180	1.000
Nerve sparing	None	0	0	1.000
	Unilateral	0	0	
	Bilateral	6	14	
PW (g)		44	43	0.455
pT stage	pT2	4	2	0.329
	≥pT3	12	2	
PSM	pT2	0 (0%)	2 (16.7%)	1.000
	≥pT3	2 (100%)	1 (50%)	1.000
Complications	≥CD grade3	0	1	1.000
Urinary continence				
Pad-free	Immediate	3 (50%)	9 (64.3%)	1.000
1 pad/day	Immediate	6 (100%)	11 (78.6%)	0.521
Erectile function	Immediate	0	0	1.000

PSA: prostate-specific antigen; BMI: body mass index; EBL: estimated blood loss; RM: resection margin; PW: prostate weight; PSM: positive surgical margin; CD: Clavien–Dindo. Data are presented in median value.

**Table 2:** Details of the clinical and surgical outcomes of the initial 6 cases of rs-RARP using hinotori platform.

No.	Age (yrs.)	PSA (ng/mL)	BMI (kg/m^2^)	CT (min)	Pad-Free		1 Pad/Day		Erectile Function		RM	PW (g)	pT	Complications
					Immediate	3 mo	Immediate	3 mo	Immediate	3 mo				
1	78	3.2	20.2	292	+	+	+	+	−	−	+	55	3	
2	76	10.2	23.8	286	−	+	+	+	−	−	−	32	2	Neuropathy *
3	62	6.4	22.7	260	+	+	+	+	−	−	−	52	2	
4	77	5.6	22.5	288	−	NA	+	+	−	−	−	38	2	
5	60	3.8	20.8	402	+	NA	+	+	−	+	−	50	2	
6	62	8.1	20.8	273	−	NA	+	+	−	+	+	32	3	

PSA: prostate-specific antigen; BMI: body mass index; CT: console time; mo: month; RM: resection margin; PW: prostate weight. * Neuropathy of the left upper extremity occurred. However, the patient recovered within 3 days.

## Data Availability

Data used in this study can be available with a reasonable request to the corresponding author and acceptance from both the corresponding author and MDPI.

## Supplementary Materials

The following supporting information can be downloaded at https://drive.google.com/file/d/1k9E1yxQH3twAcSavzv3bih6aiHUTIbzb/view, Video S1: Transection of the peritoneum. Video S2: Dissection of the vas deferens and seminal vesicles. Video S3: Bladder neck dissection. Video S4: Everting suture of the bladder neck.
